# Key ultrasound predictors in the prenatal assessment of congenital pulmonary airway malformation: a single-center experience

**DOI:** 10.3389/fped.2025.1555539

**Published:** 2025-05-16

**Authors:** Isabella Fabietti, Alice Novak, Laura Valfrè, Chiara Vassallo, Domenico Umberto De Rose, Milena Viggiano, Andrea Conforti, Chiara Iacusso, Marco Bonito, Pietro Bagolan, Leonardo Caforio

**Affiliations:** ^1^ Clinical Area of Fetal, Neonatal and Cardiological Sciences and Research Area of Perinatal Medicine – “Bambino Gesù” Children's Hospital IRCCS, Rome, Italy; ^2^ Obstetrics and Gynecology Unit, Maternal and Child Department, San Pietro Fatebenefratelli Hospital, Rome, Italy; ^3^ Department of Systems Medicine, University of Rome “Tor Vergata”, Rome, Italy

**Keywords:** congenital pulmonary airway malformation, CPAM, CVR, O/E LHR, MSA, fetal surgery, newborn

## Abstract

**Background:**

This study assesses the sensitivity and specificity of congenital pulmonary airway malformation (CPAM) Volume Ratio (CVR) in predicting the need for fetal therapy (FT) and explores the role of additional ultrasound indicators, including the Observed/Expected Lung to Head Ratio (O/E LHR) and Mediastinal Angle Shift (MSA), in improving FT prediction.

**Methods:**

We retrospectively studied all CPAM cases referred to our Center from 2018 to 2022. FT was provided at any CVR value in cases of hydrops, rapid lesion growth, or polyhydramnios. The worst CVR, O/E LHR, and MSA values between 20 and 28 weeks of gestation were analyzed.

**Results:**

Among 62 CPAM cases, 56.4% right-sided and 43.5% left-sided. Hydrops occurred in 5 cases, all right-sided. FT was required in 14 cases (11 receiving steroids and 3 thoraco-amniotic shunt). CVR was significantly higher in the FT group compared to the non-FT group (*p* < 0.0001), with an optimal cut-off of 1.25 (Sn 100%; Sp 89.6%) for predicting FT. The O/E LHR was significantly lower in the FT group (mean 44.8 vs. 58.3; *p* = 0.0046, AUC 0.75), with a Sn of 84% and Sp of 62%. MSA was significantly higher in the FT compared to the non-FT group (*p* < 0.0001), with a threshold of 13.3° providing high Sn (92.8%) and Sp (89.3%) for predicting FT.

**Conclusions:**

CVR is the most reliable predictor of the need for FT, even at lower thresholds. MSA can effectively complement CVR in predicting FT, and using multiple parameters may improve parental counseling and identify cases needing closer monitoring.

## Introduction

Congenital pulmonary airway malformation (CPAM) is the most common lesion of the fetal lung and generally has a favorable outcome in most cases. This condition typically increases in size between 18 and 26 weeks of gestation and subsequently, after a plateau, either stops growing or decreases in size (1, 2). Conversely, in some cases, the mass volume increases, leading to mediastinal shift, polyhydramnios, and hydrops, with a high risk of fetal failure and death (3). These fetuses can be treated with medical or surgical prenatal intervention depending on the type of lesion (maternal betamethasone for microcystic/solid lesions and fetal thoraco-amniotic shunt for macrocystic lesions) (4–6). Currently, the CPAM Volume Ratio (CVR) is the best prognostic index correlating with the risk of fetal hydrops for values greater than 1.6 (7). However, no clear international consensus has been reached to define the optimal CVR thresholds, as some studies have shown good predictive power even at values less than 1.6 for severe perinatal outcomes such as fetal and neonatal death, need for prenatal therapy, and need for postnatal respiratory assistance (3, 8–10). Similarly, other ultrasound (US) predictors of perinatal outcomes have been investigated with controversial results (10, 11). The aim of this study was to evaluate the sensitivity and specificity of CVR values in our population to predict the need for fetal therapy. The secondary aim was to investigate the role of other ultrasound indicators of lung hypoplasia and mediastinal shift severity, such as the lung-to-head ratio (LHR) and mediastinal shift angle (MSA), to improve the prediction of fetal treatment.

## Materials and methods

Between 2018 and 2022 all cases of CPAM referred at our fetal medicine and surgery center were prospectively collected. Exclusion criteria were multiple pregnancies and/or the presence of other associated fetal anomalies. A detailed ultrasound evaluation (General Electric Voluson E8) was performed by a scheduled fetal medicine specialist. CVR, O/E LHR and MSA were longitudinally measured as follows:

**CVR**: length×height×width × 0.52 divided by the fetal head circumference correcting for gestational age (7).

**LHR**: tracing method with values were expressed as observed/expected LHR (O/E LHR) for the right or left lung using published formulas (12).

**MSA**: We previously reported the normal range being between 17° and 24° in cases with left isolated congenital diaphragmatic hernia (CDH) (13). As we intended to extend its use to both left and right CPAMs, we normalized the degree values to obtain comparable measurements between the two sides of the defect. Considering the normalized normal range as 0, the degrees of mediastinal shift were then calculated by evaluating the deviation of the mediastinum position from 0. A value of 1 was attributed for each degree of shift, both to the right (in cases of left CPAM) and to the left (in cases of right CPAM).

Fetal therapy was administered at any CVR value in cases of hydrops and/or rapid growth of the pulmonary lesion and/or polyhydramnios. Maternal corticosteroids were given for microcystic lesions (two doses of intramuscular betamethasone 12 mg separated by 24 h, potentially repeated for a maximum of three cycles in cases of rapid lesion growth), whereas thoraco-amniotic shunting was performed for macrocystic CPAM (Harrison fetal bladder stent set, Cook Medical, Bloomington, IN, USA).

The worst value of CVR, observed-to-expected lung-to-head ratio (O/E LHR), and mediastinal shift angle (MSA) obtained between 20 and 28 weeks of gestation was considered and recorded for the analysis. For each ultrasound (US) predictor, the Receiver Operating Characteristic (ROC) curve was obtained to assess diagnostic performance and identify an optimal threshold value for fetal therapy. For each predictor, the Mann–Whitney test was performed to compare right and left cases.

Finally, a linear regression analysis was used to compare MSA values and CVR at the same gestational age. Statistical analysis was performed using GraphPad Prism version 10.0.0 for Windows (GraphPad Software, Boston, Massachusetts, USA, http://www.graphpad.com). Statistical significance was set at a *P*-value less than 0.05.

Considering the retrospective nature of the analysis, the current study did not require the regulatory approval of the local Ethics Committee according to current national legislation, but a notification was sent.

The data that support the findings of this study are available on request from the corresponding author, [IF]. The data are not publicly available as their containing information that could compromise the privacy of research participants.

## Results

A total of 62 fetuses with prenatal diagnosis of CPAM were evaluated during the study period. Characteristics of population are summarized in Table 1.

**Table 1:** Characteristics of study population.

	All CPAM	Left CPAM	Right CPAM
Number of cases	62	27	35
Maternal age median (range)	33 (18–41)	33 (25–38)	33 (18–41)
IVF *n* (%)	2 (3.2%)	1 (3.7%)	1 (2.9%)
GA at referral median (range)	24.0 (17.6–34.6)	22.3 (20.3–34.6)	23.2 (17.6–31.8)
CPAM Type *n* (%)			
1	12 (19.4)	4 (14.8)	8 (22.8)
2	44 (70.9)	21 (77.7)	23 (65.7)
3	5 (9.7)	2 (7.4)	4 (11.4)
At referrral CVR median (range)	0.69 (0.16–4.60)	0.57 (0.38–2.42)	0.73 (0.16–4.60)
Max CVR median (range)	0.84 (0.20–6.20)	0.81 (0.86–2.56)	0.84 (0.20–6.20)
Hydrops n(%)	5 (8)	0	5 (14.2)
Fetal therapy *n* (%)	14 (22.6)	4 (14.8)	10 (28.6)
Medical *n* (%)	11 (17.7)	4 (14.8)	7 (20.0)
Surgical *n* (%)	3 (4.8)	0	3 (8.6)
GA at birth median (range)	39 (31.3–41)	39 (36–41)	39 (31,3–41)
Birthweight in g Mean (SD)	3,264.2 (435)	3,168.0 (434)	3,335.4 (428)
Male newborns *n* (%)	35 (56)	18 (66.6)	17 (48.5)
Mortality *n* (%)	1 (0.01)	0	1 (0.03)

CPAMs were right-sided in 35 cases (56.4%) and left-sided in 27 cases (43.5%). Five cases (8%) with fetal hydrops were observed, all in the right CPAM group. In 14 cases (22.5%), fetal therapy was required, with steroid administration in 11 cases (78.5%) and thoraco-amniotic (TA) shunt placement in 3 cases (21.4%).

**Figure 1:**
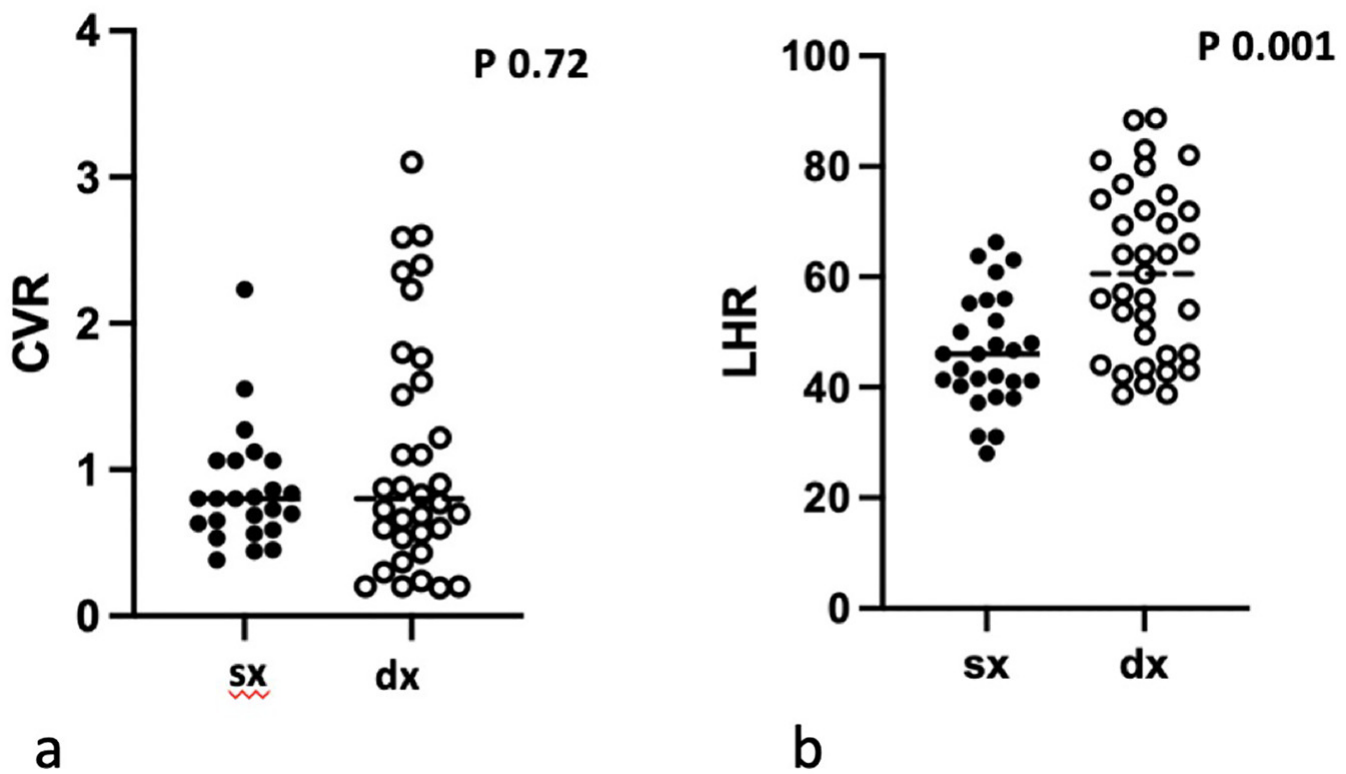
Distribution of CVR values between right and left CPAM **(a)** and O/E LHR values between right and left CPAM **(b)**.

Overall, fetal therapy was required in 22.4% (11/49) of microcystic lesions and in 25% (3/12) of macrocystic lesions, respectively. CVR was significantly higher in the fetal therapy group compared with the non-therapy group (*p* < 0.0001). No difference was observed when comparing CVR in left and right CPAMs (Figure 1a). The best cut-off value for CVR was 1.25, with a sensitivity of 100% and a specificity of 89.6%.

**Figure 2:**
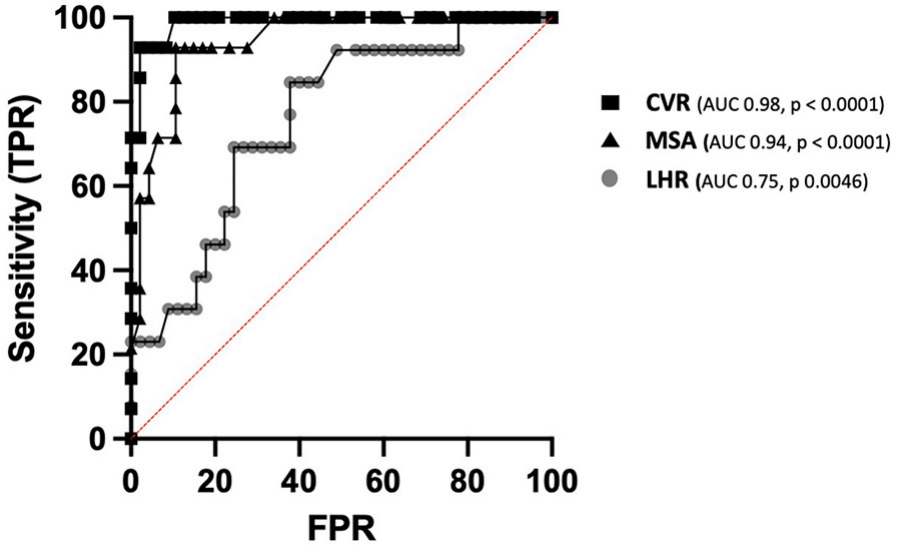
ROC curve of CVR, MSA and LHR sensitivity and sensibility in prediction of fetal therapy.

The O/E LHR was significantly lower in the fetal therapy group compared with the no-therapy group (mean 44.8 vs. 58.3, respectively, *p* = 0.0046, area under the curve 0.75). Additionally, O/E LHR was lower in left CPAMs compared to right CPAMs (mean 46 vs. 60.5, respectively, *p* = 0.0001) (Figure 1b). O/E LHR had a sensitivity of 84% and a specificity of 62%, with the best cut off value at 53.5. Receiver Operating Characteristic (ROC) curves are shown in Figure 2.

**Figure 3:**
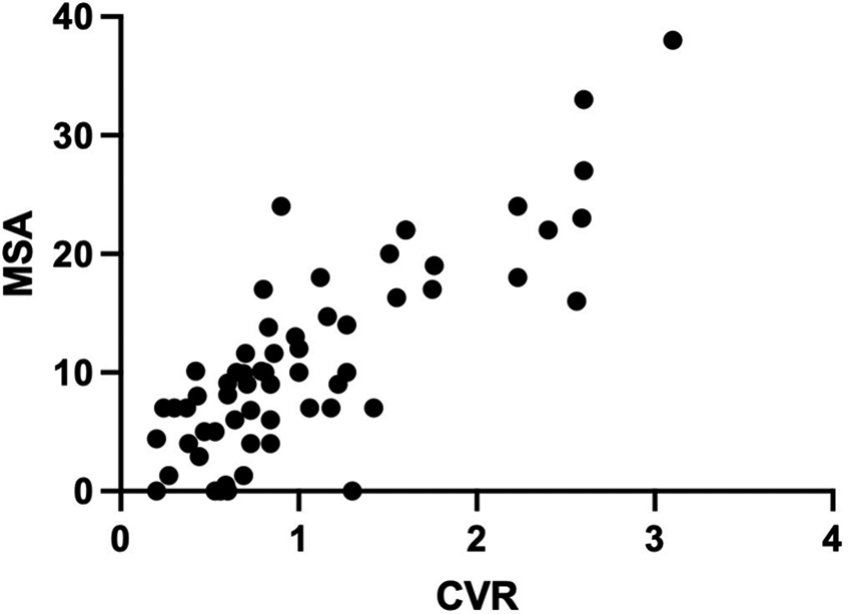
Linear regression: comparison between MSA and CVR distributions.

The MSA was significantly higher in the fetal therapy group (mean 21.7) compared with the no-therapy group (mean 8.03) (*p* < 0.0001), with no significant difference between right and left CPAMs (*p* = 0.18). MSA showed good sensitivity (92.8%) and specificity (89.3%) for predicting the need for fetal therapy, with a threshold value of 13.3 degrees [*p* < 0.0001, area under the curve (AUC) 0.94]. A significant linear regression was observed between CVR and MSA (*p* < 0.0001, r^2 = 0.67) (Figure 3).

## Discussion

In this study, the primary role of CVR in predicting and identifying cases with a higher risk of developing fetal hydrops and the need for fetal surgery was confirmed. Several recent studies have assessed the utility of CVR thresholds even lower than 1.6 in predicting perinatal outcomes such as fetal and neonatal death, the need for prenatal therapy, and postnatal respiratory assistance (8–10). Our results support these findings, demonstrating that CVR is the best tool to predict the need for fetal therapy with excellent sensitivity and specificity, even at a threshold value of 1.25.

Among the other evaluated predictors, the O/E LHR is considered the best prognostic indicator in the prenatal assessment of congenital diaphragmatic hernia (CDH) to evaluate the severity of contralateral lung hypoplasia and the related survival rate (12). In left-sided CDH, an O/E LHR value of less than 25% is associated with a poor prognosis at birth, with a survival rate of 20% (14). The mass effect of CPAMs on fetal lungs is the physiological rationale that led us to evaluate O/E LHR in fetuses with CPAM as well. Hellmund et al. analyzed CVR in CPAM, showing lower sensitivity when compared with CVR in CDH (10). Our data confirm this result, likely due to the completely different pathogenesis and natural history of these conditions. The type of mass effect on the contralateral lung in CDH is due to the persistence of many abdominal organs (bowel, stomach, and liver) in the thorax throughout gestation (10), as well as the hypothesis of primary lung damage in CDH (dual-hit hypothesis) and different pulmonary hypoplasia observed in the nitrofen model of congenital diaphragmatic hernia (15). The long-lasting herniation during gestation may make the hypoplasia of the ipsilateral lung more severe, resulting in a worse O/E LHR.

In CPAM, we speculate that the mass effect on the contralateral lung is likely lower than in CDH due to the difference in the degree of compression by the abnormal lung tissue and the potential for natural regression reported in CPAM. The natural history of CPAM, characterized by progressive regression of the mass usually after 27–29 weeks of gestation (1, 10) might explain the difference in the predictive value of O/E LHR for severe cases of CPAM.Our group first described the significant value of the mediastinal shift angle (MSA) in predicting the outcome of left severe isolated congenital diaphragmatic hernia (CDH) (13). Shulman et al. (16) first identified a significant correlation between MSA and CVR in CPAM, suggesting its good performance in predicting adverse perinatal outcomes. However, the authors used different methods to measure MSA for left and right CPAMs, which prevents direct comparison of this parameter between the two types of lesions.

Our data show that MSA is a good predictor of the need for fetal therapy. Although the sensitivity of CVR was greater than that of MSA (100% vs. 92.8%, respectively), the specificity of both tools was similar (89.5% for CVR and 89.3% for MSA). The significant linear correlation between CVR and MSA suggests that MSA has good applicability in clinical management and parental counseling for CPAM.

Similarly, a recent study reported that MSA, measured by fetal MRI, has the potential to replace CVR for evaluating the severity of CPAM (11). In our population, all cases of hydrops occurred in right-sided CPAMs, raising the hypothesis that anatomical and/or hemodynamic mechanisms could play a significant role. Specifically, venous systemic returns and the thoracic duct, which are located in the right hemithorax for at least half of their course, might be compressed or deviated by the lung lesion, potentially leading to hydrops. To validate this hypothesis, further data are needed.

Our results may stem from the decision to intervene even before hydrops appearance, in cases of rapid growth of the lesion or polyhydramnios. This opens up the discussion about a potentially different threshold for the need for fetal intervention, which could be useful for guiding short-term follow-up and parental counseling.

Further studies involving larger case series are needed to compare the risk of prenatal intervention between fetuses with CVR values between 1.25 and 1.6 and those with CVR values greater than 1.6.

To conclude, in the prenatal assessment of both right and left CPAM, CVR remains the best predictor of the need for fetal therapy, even at thresholds lower than 1.6. MSA is a simple ultrasound value that could be used in conjunction with CVR to predict the need for fetal therapy. Additionally, right-sided lesions should be considered an extra risk factor for fetal hydrops. Although CPAM often has a good perinatal prognosis, evaluating multiple indicators can better identify and manage cases with a high-risk prenatal natural history. Utilizing more than one parameter may improve parental counseling and help identify cases that require closer ultrasound surveillance.

## Data Availability

The raw data supporting the conclusions of this article will be made available by the authors, without undue reservation.

## References

[1] Adzick NS. Management of fetal lung lesions. Clin Perinatol. (2009) 36(2):363–76. 10.1016/j.clp.2009.03.001 19559325

[2] Cavoretto P Molina F Poggi S Davenport M Nicolaides KH. Prenatal diagnosis and outcome of echogenic fetal lung lesions. Ultrasound Obstet Gynecol. (2008) 32:769–83. 10.1002/uog.6218 18956429

[3] Sileo FG Alameddine S Iaccarino DA Di Mascio D Giuliani GA Bertucci E Outcome of fetal congenital pulmonary malformations: a systematic review and meta-analysis. J Perinat Med. (2024) 52(5):457–66. 10.1515/jpm-2024-0017 38651628

[4] Peranteau WH Boelig MM Khalek N Moldenhauer JS Martinez-Poyer J Hedrick HL Effect of single and multiple courses of maternal betamethasone on prenatal congenital lung lesion growth and fetal survival. J Pediatr Surg. (2016) 51(1):28–32. 10.1016/j.jpedsurg.2015.10.018 26526208

[5] Curran PF Jelin EB Rand L Hirose S Feldstein VA Goldstein RB Prenatal steroids for microcystic congenital cystic adenomatoid malformations. J Pediatr Surg. (2010) 45(1):145–50. 10.1016/j.jpedsurg.2009.10.025 20105595

[6] Peranteau WH Adzick NS Boelig MM Flake AW Hedrick HL Howell LJ Thoracoamniotic shunts for the management of fetal lung lesions and pleural effusions: a single-institution review and predictors of survival in 75 cases. J Pediatr Surg. (2015) 50(2):301–5. 10.1016/j.jpedsurg.2014.11.019 25638624

[7] Crombleholme TM Coleman B Hedrick H Liechty K Howell L Flake AW Cystic adenomatoid malformation volume ratio predicts outcome in prenatally diagnosed cystic adenomatoid malformation of the lung. J Pediatr Surg. (2002) 37:331–8. 10.1053/jpsu.2002.30832 11877643

[8] Kane SC Ancona E Reidy KL Palma-Dias R. The utility of the congenital pulmonary airway malformation-volume ratio in the assessment of fetal echogenic lung lesions. A Systematic Review Fetal Diagn Ther. (2020) 47(3):171–81. 10.1159/000502841 31593968

[9] Yong PJ Von Dadelszen P Carpara D Lim K Kent N Tessier F Prediction of pediatric outcome after prenatal diagnosis and expectant antenatal management of congenital cystic adenomatoid malformation. Fetal Diagn Ther. (2012) 31(2):94–102. 10.1159/000331936 22310905

[10] Hellmund A Berg C Geipel A Bludau M Heydweiller A Bachour H Prenatal diagnosis and evaluation of sonographic predictors for intervention and adverse outcome in congenital pulmonary airway malformation. PLoS One. (2016) 11(3):e0150474. 10.1371/journal.pone.0150474 26978067 PMC4792474

[11] Tsukamoto J Miyazaki O Saito Y Irahara S Okamoto R Miyasaka M Assessment of mediastinal shift angles in congenital pulmonary airway malformation: a new fetal magnetic resonance imaging indicator of congenital lung disease. Pediatr Radiol. (2024) 54(5):715–24. 10.1007/s00247-024-05852-5 38285191

[12] Jani JC Peralta CF Nicolaides KH. Lung-to-head ratio: a need to unify the technique. Ultrasound Obstet Gynecol. (2012) 39:2–6. 10.1002/uog.11065 22213615

[13] Romiti A Viggiano M Conforti A Valfré L Ravà L Ciofi Degli Atti M Ultrasonographic assessment of mediastinal shift angle (MSA) in isolated left congenital diaphragmatic hernia for the prediction of postnatal survival. J Matern Fetal Neonatal Med. (2020) 33(8):1330–5. 10.1080/14767058.2018.1517329 30153757

[14] Jani J Nicolaides KH Keller RL Benachi A Peralta CF Favre R Observed to expected lung area to head circumference ratio in the prediction of survival in fetuses with isolated diaphragmatic hernia. Ultrasound Obstet Gynecol. (2007) 30(1):67–71. 10.1002/uog.4052 17587219

[15] Keijzer R Liu J Deimling J Tibboel D Post M. Dual-hit hypothesis explains pulmonary hypoplasia in the nitrofen model of congenital diaphragmatic hernia. Am J Pathol. (2000) 156(4):1299–306. 10.1016/S0002-9440(10)65000-6 10751355 PMC1876880

[16] Shulman R Sparks TN Gosnell K Blat C Norton ME Lee H Fetal congenital pulmonary airway malformation: the role of an objective measurement of cardiomediastinal shift. Am J Perinatol. (2019) 36(3):225–32. 10.1055/s-0038-1669909 30199894 PMC6372337

